# Editorial: Quantitative Susceptibility Mapping in Neurodegeneration

**DOI:** 10.3389/fnins.2021.724550

**Published:** 2021-07-22

**Authors:** Fuhua Yan, Naying He, E. Mark Haacke

**Affiliations:** ^1^Department of Radiology, Ruijin Hospital, Shanghai Jiao Tong University School of Medicine, Shanghai, China; ^2^Department of Biomedical Engineering and Radiology, Wayne State University, Detroit, MI, United States

**Keywords:** quantitative susceptibility mapping, neurodegenerative disease, brain iron, deep grey matter, paramagnetic and diamagnetic materials

Magnetic resonance imaging offers a wide variety of contrast mechanisms to study soft tissue in the body. Some common methods include: T1 weighted (T1W) imaging, T2 weighted (T2W) imaging, spin density imaging, diffusion weighted imaging (DTI), flow imaging, and susceptibility weighted imaging (SWI). Using the complex data from the SWI scans, it is possible to produce magnetic source images using a novel reconstruction method that takes the phase data and reconstructs what is referred to as a quantitative susceptibility map (QSM). The phase images represent local frequency changes caused by local changes in the magnetic field which in turn are introduced because of the presence of a magnetic source. Some of the familiar paramagnetic materials include deoxygenated blood, hemosiderin and ferritin while some of the more familiar diamagnetic materials include calcium deposits (in the pineal gland, choroid plexus, basal ganglia, and in vessel wall), calcium in bone, and myelinated tissue. A number of diseases cause local changes in iron content including: Parkinson's disease (PD), Huntington's disease, Friedrich's Ataxia and keratopathies in the brain and hemochromatosis in the liver. The ability to measure iron has clear implications if iron is involved in the pathophysiology or correlates with disease state, but it must be remembered that iron also changes as a function of age (Li Y. et al.). Also, measuring oxygen saturation would be key in stroke and potentially in dementia and other neurodegenerative diseases as well. Although QSM has been studied for more than 15 years (Haacke et al., 2015), it has taken some time to find its way into clinical studies. The goal of this special issue was to point towards future clinical applications of QSM. In this issue, iron was studied in patients with: Parkinson's disease, Alzheimer's disease, mild cognitive impairment, diabetes mellitus, occlusion of the middle cerebral artery, and Wilson's disease.

A systematic overview of the role of iron in various neurodegenerative diseases is given by Ravanfar et al. They reviewed 80 records by searching MEDLINE, Embase, Scopus, and PsycInfo databases to determine which diseases had at least one structure that demonstrated increased iron content. They found that different parts of the deep grey matter were affected for different diseases (including Alzheimer's disease, Parkinson's disease, amyotrophic lateral sclerosis, Wilson's disease, Huntington's disease, Friedreich's ataxia, etc.). In some disorders, the increased magnetic susceptibility correlated with disease duration and severity of clinical features. They conclude that QSM is a promising tool to study changes in brain iron.

More specifically, Song et al. investigated iron deposition in PD patients associated with levodopa-induced dyskinesia (LID). They evaluated the ability of substantia nigra (SN) iron content to discriminate PD with and without LID. Interestingly, they found that the susceptibility of the SN for PD patients with LID was higher than that in PD patients without LID. Liu X. et al. evaluated data for both QSM and DTI in an Alzheimer disease cohort. They studied iron in the basal ganglia and found that the susceptibility of the right caudate nucleus in patients with AD correlated with cognitive scores. Several other diffusion measures also correlated positively with the magnetic susceptibility of the right caudate nucleus in patients with Alzhiemer's disease. Their findings revealed that the magnetic susceptibility of the caudate nucleus may be an MRI-based biomarker of the cognitive dysfunction of AD and abnormal excessive iron distribution in the basal ganglia had adverse effects on the diffusion properties of the white matter. Jin et al. studied iron in the mammillary body for a group of mild cognitively impaired (MCI) patients. They did not find significant correlations with the patient's cognitive status but they were able to establish a normative range of iron in the mammillary bodies. They did find that susceptibility was elevated in the right mammillary body in MCI patients compared to healthy controls and that there was a small but significant reduction in volume with age for the MCI group. Li J. et al. studied the brain iron changes in patients with diabetes mellitus. They evaluated brain iron accumulation in type 2 diabetes mellitus (T2DM) patients with executive function decline using a voxel-based quantitative susceptibility. They found patients with T2DM had higher iron than healthy controls in the basal ganglia and frontal lobe. There appeared to be some correlation with cognitive testing and iron in the thalamus. Du et al. studied patients with middle cerebral artery occlusion. They found changes of iron in the basal ganglia and evaluated the ability of iron content to predict the presence of middle cerebral artery occlusion. Yuan et al. studied paramagnetic metal accumulation in the deep grey matter in patients with Wilson's Disease. Not only did they find increases in susceptibility throughout the deep grey matter, but they also found a reduction in their volumes. Of clinical interest, the Unified Wilson's Disease Rating (UWDRS) neurological subscores were positively correlated with the susceptibility values of all examined deep grey matter structures. An area of great interest is measuring oxygen saturation, particularly for stroke patients. Liu Y. et al. evaluated many of the veins associated with specific structures and found that there was a significant negative correlation with a number of cognitive measures in patients with AD. This decreasing cerebral venous oxygen level suggests that there is a reduced flow but still high cerebral metabolic rate of oxygen utilisation.

On a more technical front, Gharabaghi et al. presented a new approach for multi-echo QSM using a method referred to as Strategically Acquired Gradient Echo (STAGE) Imaging using a structural constrained approach. They evaluated both simulated data and human data and showed high accuracy relative to other methods. This approach helps take advantage of the benefits of both short echoes and long echoes without constraining one to the other. Finally, a key issue for more broad use of QSM is its reliability across sites and across manufacturers with different resolutions. Li Y. et al.  studied a large population of over 600 healthy adults, ranging from 20 to 90 years old, across 3 sites, 3 manufacturers and two field strengths. Generally, they found that the iron growth as a function of age was well-replicated across all the variables although smaller structures tended to have the largest errors. They also found that volume was generally negatively correlated with age. As usual they found that most structures significantly increased their iron content with age and that this was even more evident when the local high iron content regions were assessed. This iron dependence with age is critical to catalogue when studying any neurodegenerative disease with suspected increases in iron content since the data needs to be age corrected.

In summary, there has been great progress in using QSM to study neurodegenerative diseases. Despite the different QSM methods that are available today, as long as the same approach is used for a given study and as long as the age-related iron effect is removed, this method offers a new means to study the pathophysiology of neurodegenerative diseases that complements and generally improves upon the use of ${\text{T}}_{2}^{*}$ measures of iron content (Ghassaban et al., 2018; Yan et al., 2018). The papers presented in this special issue show the potential of QSM to enhance diagnosis of patients with neurodegenerative disease. As QSM becomes more broadly accepted, no doubt other clinical applications of measuring susceptibility will emerge.

## Author Contributions

FY, NH, and EH wrote the manuscript. All authors contributed to the article and approved the submitted version.

## Conflict of Interest

The authors declare that the research was conducted in the absence of any commercial or financial relationships that could be construed as a potential conflict of interest.
